# Green Production of Cladribine by Using Immobilized *2′*-Deoxyribosyltransferase from *Lactobacillus delbrueckii* Stabilized through a Double Covalent/Entrapment Technology

**DOI:** 10.3390/biom11050657

**Published:** 2021-04-29

**Authors:** Cintia Wanda Rivero, Natalia Soledad García, Jesús Fernández-Lucas, Lorena Betancor, Gustavo Pablo Romanelli, Jorge Abel Trelles

**Affiliations:** 1 Laboratory of Sustainable Biotechnology (LIBioS), National University of Quilmes, Bernal B1876BXD, Argentina; garcia.nataliansg@gmail.com; 2 National Scientific and Technical Research Council (CONICET), Ciudad Autónoma de Buenos Aires C1425FQB, Argentina; 3 Applied Biotechnology Group, Biomedical Science School, Universidad Europea de Madrid, 28670 Villaviciosa de Odón, Spain; JESUS.FERNANDEZ2@universidadeuropea.es; 4 Research Group in Natural and Exact Sciences, GICNEX, Universidad de la Costa, CUC, Barranquilla 080002, Colombia; 5 Biotechnology Laboratory, Faculty of Engineering, Universidad ORT Uruguay, Montevideo 11100, Uruguay; betancor@fi365.ort.edu.uy; 6 Center for Research and Development in Applied Sciences, National University of La Plata, La Plata B1900AJK, Argentina; gpr@quimica.unlp.edu.ar

**Keywords:** biomimetic silica, enzyme immobilization, glutaraldehyde, entrapment, calcium alginate, antineoplastic drug

## Abstract

Nowadays, enzyme-mediated processes offer an eco-friendly and efficient alternative to the traditional multistep and environmentally harmful chemical processes. Herein we report the enzymatic synthesis of cladribine by a novel 2′-deoxyribosyltransferase (NDT)-based combined biocatalyst. To this end, *Lactobacillus delbrueckii* NDT (*Ld*NDT) was successfully immobilized through a two-step immobilization methodology, including a covalent immobilization onto glutaraldehyde-activated biomimetic silica nanoparticles followed by biocatalyst entrapment in calcium alginate. The resulting immobilized derivative, SiG_PEI 25000_-*Ld*NDT-Alg, displayed 98% retained activity and was shown to be active and stable in a broad range of pH (5–9) and temperature (30–60 °C), but also displayed an extremely high reusability (up to 2100 reuses without negligible loss of activity) in the enzymatic production of cladribine. Finally, as a proof of concept, SiG_PEI 25000_-*Ld*NDT-Alg was successfully employed in the green production of cladribine at mg scale.

## 1. Introduction

Cladribine (2-chloro-2′-deoxy-β-D-adenosine) is an FDA approved drug for the treatment of hairy cell leukemia [1]. It has also recently received EMA approval for relapsing–remitting multiple sclerosis (RRMS) treatment [2].

Traditionally, cladribine is synthesized by chemical methods that require multiple reaction steps, the use of organic solvents, and removal of protecting groups, causing the accumulation of racemic mixtures that affect further purification [3,4]. However, the use of bioprocesses catalyzed by whole cells or enzymes has emerged as a green synthetic alternative with multiple advantages, such as mild reaction conditions, high efficiency, and regio-, stereo-, enantioselectivity [5,6,7,8,9,10,11,12,13,14,15]. In this sense, the transglycosylation reaction catalyzed by nucleoside phosphorylases (NPs) or 2′-deoxyribosyltransferases (NDTs) is the most studied biocatalytic methodology for the synthesis of nucleoside analogs (NAs) up to date [5,6,7,8,9,10,11,12,13,14,15].

Several factors such as low operational stability, short availability, the high cost of recombinant enzymes, and difficult recovery have hindered the use of enzymes for industrial applications [16]. Some of these drawbacks can be overcome by the immobilization of the biocatalyst, which can improve enzyme properties, such as activity and specificity. Additionally, it enables an easy product recovery and also increases the half-life of enzymes, and allows for biocatalyst reusability [17,18,19,20].

In this regard, the use of silica-based matrixes in enzyme immobilization has started to attract interest due to its nontoxicity, excellent biocompatibility, and stability over a wide range of pressure, pH, and temperature [21,22,23,24]. Under mild conditions silica formation in biogenic systems, is mediated by cationic proteins and peptides, but it can also be mediated by a range of simple polyamine molecules [25,26]. Furthermore, entrapment methods are also widely used for enzyme immobilization, and one of the most common supports is alginate, a natural anionic biopolymer usually obtained from brown seaweed [27]. Because of its biocompatibility, nontoxicity, and relatively low cost, alginate has been extensively used for pharmaceutical and medical applications [28,29].

**Figure 1:**
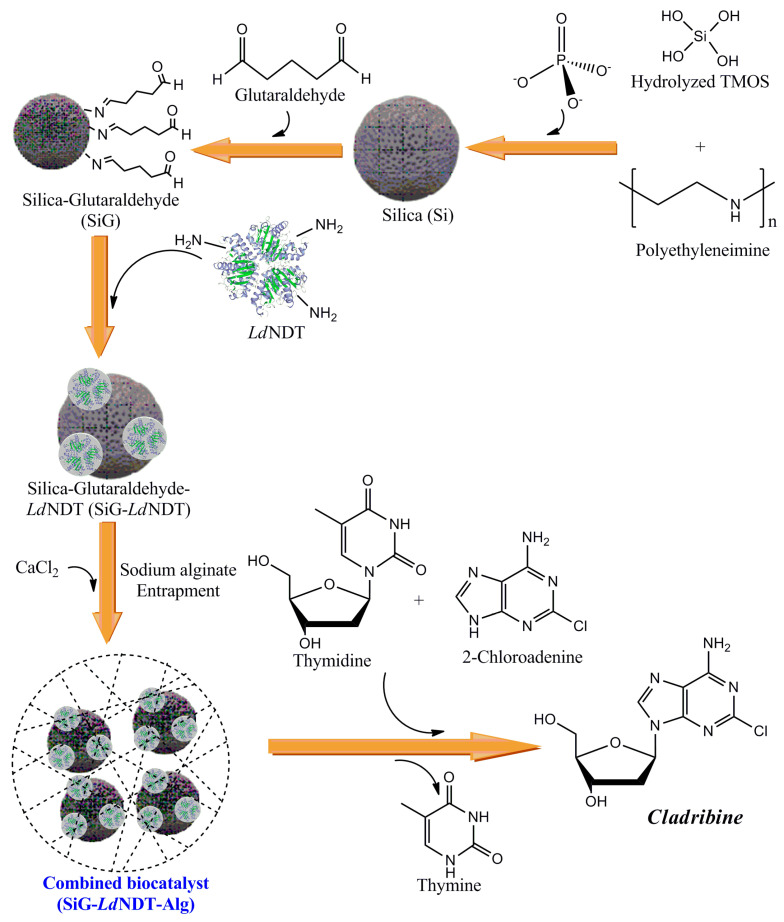
Schematic representation of the combined immobilization procedure, carried out in this work to obtain cladribine, through the development of a highly stabilized biocatalyst. TMOS: tetramethylorthosilicate, PEI: Polyethyleneimine and *Ld*NDT: Recombinant NDT from *Lactobacillus delbrueckii.*

Herein we show the development of a highly stabilized biocatalyst for the production of cladribine based on immobilization of 2′-deoxyribosyltransferase from *Lactobacillus delbrueckii* (*Ld*NDT) on modified silica-based matrixes through a two-step immobilization methodology, including a covalent immobilization onto glutaraldehyde-activated biomimetic silica nanoparticles followed by biocatalyst entrapment in calcium alginate (Figure 1). Biochemical characterization of the immobilized derivatives, including the effect of pH and temperature on enzyme activity and stability, as well as the biocatalysts reusability, led us to select an optimal biocatalyst for further scale-up. Finally, the developed biocatalyst was successfully employed in the production of cladribine at mg scale (1.8 mg) using a batch system with an airlift column.

## 2. Materials and Methods

### 2.1. Materials

Nucleosides and bases were purchased from Sigma-Aldrich (USA). Culture media compounds were obtained from Britania S.A. (CABA, Argentina). Polyethyleneimine (PEI, MW 1.200–1.300, 25.000 and 70.000) was from Sigma Aldrich (St. Louis, MI, USA). The HPLC grade solvents used were from Sintorgan S.A. (Villa Martelli, Argentina). Cyanogen bromide (CNBr) activated Sepharose beads werepurchased from GE-Healthcare (Uppsala, Sweden),tetramethylorthosilicate (TMOS) was purchased from Merck S.A. (Gernsheim, Germany) and sodium alginate was from Saporiti S.A.C.I.F.I.A (CABA, Argentina). All other reagents were of analytical grade.

### 2.2. Production of LdNDT

Type II NDT from *Lactobacillus delbrueckii* (*Ld*NDT) was produced and purified as previously reported [30,31].

### 2.3. Standard Activity Assay for LdNDT 

Biotransformation of cladribine from thymidine (Thd) and 2-chloroadenine (2-ChlAde) was selected as standard reaction to evaluate enzymatic activity. To this effect, 10 µg *Ld*NDT (free or immobilized) were added to solution containing 1.5 mM Thd and 0.5 mM 2-ChlAde in 25 mM tris-HCl buffer, pH 7.0, at 50 °C and 200 rpm shaking speed. The enzyme was inactivated by adding 100 µL of cold methanol in an ice-bath and heating for 5 min at 100 °C. After centrifugation at 10,000 rpm for 3 min, the samples were half-diluted with water and frozen at −20 °C. Nucleoside production was analyzed using HPLC to measure quantitatively the reaction products, as described below. All of the determinations were carried out in triplicate and the maximum error was less than 5%. In such conditions, one unit of enzyme (U) was defined as the amount (mg) of enzyme that produces 1 µmol/min (IU) of cladribine.

### 2.4. LdNDT Immobilization 

TMOS was hydrolyzed with 1 mM HCl in 157:1000 (*v/v*) ratio. 10% (*v/v*) solutions of PEI (Mw 1.200–1.300, 25.000 or 70.000, depending on the case) were prepared in 100 mM sodium phosphate buffer (pH 8.0).

#### 2.4.1. Biomimetic Silica Entrapment (SiBio)

For *Ld*NDT entrapment in SiBio, 2.5 mL of freshly hydrolyzed TMOS, 2.5 mL of a PEI solution (10%, *v/v*), and *Ld*NDT solution containing 230 μg/mL were mixed. Instant precipitation and subsequent enzyme entrapment wereobserved. The preparation was centrifuged at 5000 rpm for 10 min and washed with NaCl 500 mM to eliminate proteins ionically adsorbed to nanoparticle surface. Then, the SiBio biocatalyst was washed three times with 25 mM sodium phosphate buffer (pH 7.0) until use.

#### 2.4.2. Immobilization on Modified Biomimetic Silica Nanoparticles

To obtain biomimetic silica nanoparticles, 2.5 mL of freshly hydrolyzed TMOS was added to 2.5 mL of a PEI solution (10%, *v/v*) and the preparation was mixed. The nanoparticles formed were centrifuged at 5000 rpm for10 min and washed with 25 mM sodium phosphate buffer (pH 7.0).

To obtain glyoxyl modified biomimetic silica support (SiGlx), 1 g of crude silica was mixed in 0.28 mL of distilled water. Then, 32 mg of sodium hydroxide (NaOH) previously dissolved in 0.5 mL of distilled water was added and subsequently 14 mg of solid BH_4_Na was incorporated. Afterwards, 0.35 mL of glycidol was added drop by drop, leaving it for 16 h at room temperature with gentle agitation. Finally, reactive groups were activated by incubation for 2 h at room temperature with 15 mL of 10 mM NaIO_4_ solution. It was recovered by centrifugation at 5000 rpm for 10 min, rinsed with distilled water, and finally with 25 mM sodium phosphate buffer (pH 7.0). *Ld*NDT immobilization on SiGlx, was carried out at 4 °C using 0.5 g of support and 10 mL of enzyme in carbonate buffer (0.1 M; pH 10.0). The mixtures were centrifuged for 15 min at 5000 rpm and the supernatant was recovered for protein quantification several times to determine the percentage of immobilization [32]. Finally, residual reactive groups were inactivated by addition of BH_4_Na (1 mg/mL).

Glutaraldehyde-modified biomimetic silica nanoparticles (SiG) were synthesized using, 1 g silica which was incubated with 20 mL of a glutaraldehyde solution (15%, *v/v* in 25 mM sodium phosphate buffer, pH 7.0) at 25 °C and agitation by inversion for overnight. The SiG support was centrifuged and washed three times with 25 mM sodium phosphate buffer (pH 7.0). For *Ld*NDT immobilization, 0.5 g of SiG was mixed with 10 mL of enzyme solution in 25 mM sodium phosphate buffer (pH 7.0) and incubation was performed at 4 °C. The mixtures were centrifuged for 15 min at 5000 rpm and the supernatant was recovered for protein quantification several times to determine the percentage of immobilization [32].

To carry out *Ld*NDT immobilization by adsorption on the silica nanoparticles surface and subsequently coating them with glutaraldehyde (SiAdsG), 0.5 g of crude silica was incubated with 10 mL of enzyme solution in 25 mM sodium phosphate buffer (pH 7.0) at 4 °C for 16 h. After incubation, the derivative was recovered by centrifugation and mixed with glutaraldehyde 0.5% (*v/v*) in 25 mM sodium phosphate buffer (pH 7.0) at 25 °C for 1 h to allow cross-linking reaction.

#### 2.4.3. Cyanogen Bromide (CNBr) Immobilization 

For CNBr immobilization, 10 mL of purified *Ld*NDT in 25 mM sodium phosphate buffer (pH 7.0) were added to 0.5 g of previously activated support. The mixture was stirred gently for 2 h, at 4 °C and after that, the percentage of immobilization was calculated [32]. Then, the supernatant was removed and 20 mL of ethanolamine (1 M, pH 8.0) wasadded for 2 h to block the cyanogen bromide groups on the support. Finally, the derivative was washed twice with 25 mM sodium phosphate buffer (pH 7.0) and stored at 4 °C until use.

### 2.5. Biochemical Characterization of SiG_PEI25000_-LdNDT Derivative 

To assay the effect of pH and temperature on biocatalyst activity, 33 mg SiG_PEI25000_-*Ld*NDT (10 µg of immobilized *Ld*NDT) were incubated with 1 mL of reaction solution containing 0.5 mM 2-ChlAde and 1.5 mM Thd at 200 rpm during different times. The effect of pH on SiG_PEI25000_-*Ld*NDT activity was tested using sodium acetate (pH 5.0) and tris-HCl (pH 7.0 and 9.0), as reaction buffers (25 mM). Moreover, the temperature profile was assessed across a 30–60 °C interval.

### 2.6. Derivative Entrapment in Alginate

There were 0.33 g SiG_PEI25000_-*Ld*NDT derivative added to a 1 mL sodium alginate 6% (*p/v*) in physiologic solution. The mixture was added drop-wise to a 30 mL stirred solution of 0.3 M CaCl_2_ and incubated for 15 min at 25 °C [33]. The formed beads, SiG_PEI25000_-*Ld*NDT-Alg, were filtered and washed twice with 25 mM tris-HCl buffer (pH 7.0).

### 2.7. Surface Morphology Study 

Scanning electron microscopy (SEM) with energy dispersive X-ray spectroscopy (EDX) analysis was made with a Philips 505 scanning electron microscope using an accelerating voltage of 25 eV. The solid samples were metalized with gold. The chemical composition of the materials was analyzed by X-ray scattering.

### 2.8. Thermal Inactivation and pH Stability

To study the thermal stability, the obtained biocatalysts SiG_PEI25000_-*Ld*NDT and SiG_PEI25000_-*Ld*NDT-Alg were incubated at 30, 50 and 60 °C in 25 mM tris-HCl buffer (pH 7.0), for different times during periods superior than 3000 h. 

In a similar way, the effect of pH on biocatalysts stability was determined. SiG_PEI25000_-*Ld*NDT and SiG_PEI25000_-*Ld*NDT-Alg were incubated at 30 °C in the presence of 25 mM sodium acetate buffer (pH 5), 25 mM tris-HCl buffer (pH 7), or 25 mM tris-HCl buffer (pH 9) at different times during 3300 h. 

Residual activity in cladribine biosynthesis as previously described was determined. Protein release was evaluated by Bradford and the amount of protein detected in supernatant was determined.

### 2.9. Storage Stability and Operational Reusability

Storage stability was assayed by keeping SiG_PEI25000_-*Ld*NDT and SiG_PEI25000_-*Ld*NDT-Alg biocatalysts in 25 mM tris-HCl buffer (pH 7.0) at 4 °C and determining its ability in cladribine biosynthesis for 300 days, as previously described. 

Furthermore, the reusability of SiG_PEI25000_-*Ld*NDT and SiG_PEI25000_-*Ld*NDT-Alg was evaluated through successive standard cladribine biotransformations until 50% of initial activity loss or matrix integrity loss. Each reuse was performed for 15 min under previously optimized conditions. 

### 2.10. Bioprocess Scale-Up 

A scale-up bioprocess was assayed, for cladribine biosynthesis, using a batch system with an airlift column (H: 120 mm, D: 3.4 mm). Biosynthesis was carried out using 0.3 g of SiG_PEI25000_-*Ld*NDT-Alg in 10 mL of reaction medium, containing 0.5 mM 2-ChlAde and 1.5 mM Thd in 25 mM tris-HCl buffer (pH 7.0) at 30 °C.

### 2.11. Analytical Methods 

Cladribine biosynthesis was quantitatively monitored by HPLC (Dionex, Ultimate 3000, Thermo Scientific) equipped with a UV detector (UV/Vis 156, Dionex) using a Nucleosil 10 C18 100A column (10 μm, 300 mm × 4 mm). An isocratic mobile phase of water/methanol (90:10, *v/v*) and a flow rate of 1.5 mL/min were used and evaluated at 265 nm. Retention times of thymine (Thm), Thd, 2-ChlAde, and 2-ChlAdo (cladribine) were 3.3, 5.1, 12.2, and 26.8 min, respectively. Cladribine was characterized in multiple reaction monitoring (MRM) mode using an AB5000 triple quadrupole mass spectrometer (Applied Biosystems, San Jose, CA) equipped with an electrospray ionization source (ESI). The ion ESI-mass spectrum of the product showed the peak of 2-ChlAdo [M+H]^+^: 286, which were consistent with the molecular mass described for the product (average mass: 285.6873 and monoisotopic mass: 285.0629). The software Xcalibur 1.3 was used.

### 2.12. Molecular Modeling

As previously described type II NDT from *Lactobacillus leichmannii*, *Ll*NDT (PDB id 1F8X) was selected as structural template for homology modeling (98% of identity) employing Swiss-Model server. Missing amino acids in all subunits were accomplished throughout interactive molecular graphics program PyMOL [34]. Thd was manually docked into *Ld*NDT active site by means of structural best-fit superposition onto the former nucleoside. The system was prepared and energy progressively refined in TIP3P explicit solvent according to [35]. Moreover, 30 ns of unrestrained molecular dynamics simulation were performed to achieve the most suitable *Ld*NDT-Thd complex, according to a previously described protocol [36]. Resulting trajectories were processed by module implemented in AMBER16 [37].

### 2.13. Sustainability Impact

Green chemical parameters of the described bioprocesses were calculated to demonstrate mass utilization efficiency. The environmental factor (E-Factor) is a measure of the industrial environmental impact. Carbon efficiency (C-efficiency) and atom economy (A-economy) are designed as parameters to evaluate the efficiency of synthetic reactions. All the above-mentioned parameters were calculated as previously described [38].

## 3. Results and Discussion

### 3.1. Enzyme Immobilization Screening

As previously described, *Ld*NDT is able to recognize many different purine analogs and has high activity across a wide range of temperatures [31]. However, the use of soluble enzymes in biocatalysis has many limitations because of the high cost of recombinant enzymes, low stability, and the complicated downstream processing to recover the enzyme from the reaction media [39]. Based on this, enzyme immobilization emerges as an alternative to overcome all these drawbacks, favoring product recovery and improving biocatalyst reusability and stability. Therefore, *Ld*NDT was selected as a candidate for further immobilization studies. 

The use of silica nanoparticles as a support for enzyme immobilization has attracted considerable attention due to their biocompatibilty, low toxicity, and scalable availability. The silica synthesized by diatoms is an interesting starting point for enzyme immobilization since it occurs under mild conditions compatible with biological activity [26,40]. Biomimetic or bio inspired silica is thein vitrosilica formation through reactions derived or similar to those occurring in vivo. It produces nanostructured particles and is carried out at close to neutral pH and room temperature under aqueous conditions.

With this aim, *Ld*NDT immobilization was assayed onto different biomimetic aminated silica supports using several methodologies, such as biocatalyst entrapment on biomimetic silica matrix (SiBio) [41], covalent immobilization onto silica nanoparticles functionalized with glyoxyl (SiGlx) [10,42,43] or glutaraldehyde (SiG) [44,45,46], and ionic adsorption [47] onto biomimetic silica nanoparticles followed by glutaraldehyde cross-linking (SiAdsG) [42,43,46]. Moreover, as a control of the immobilization process, *Ld*NDT was also immobilized onto agarose activated with cyanogen bromide (CNBr) [48], a well-known support used for enzyme immobilization.

Due to the low immobilization percentages, together with a significant loss of catalytic activity observed when *Ld*NDT was immobilized in SiBio and SiGlx; the SiBio-*Ld*NDT and SiGlx-*Ld*NDT derivatives were discarded after the initial screening (data not shown). In contrast, the derivatives obtained after *Ld*NDT immobilization on SiG, SiAdsG and CNBr supports (SiG-*Ld*NDT, SiAdsG-*Ld*NDT, and CNBr-*Ld*NDT) displayed high immobilization percentages (50–100%), and also, a high retained activity (around 70–100%).

Since soluble *Ld*NDT was stable at alkaline pH values for a period of 30 h [30], it was not expected that pH 10 incubation onto silica functionalized with glyoxyl (SiGlx) led to this drastic loss of activity. So, too deep into this unexpected side-effect and to try to understand the effect of multipoint binding on enzyme activity, different 3D homology models of both, *Ld*NDT and complexed with Thd, were built. 

**Figure 2:**
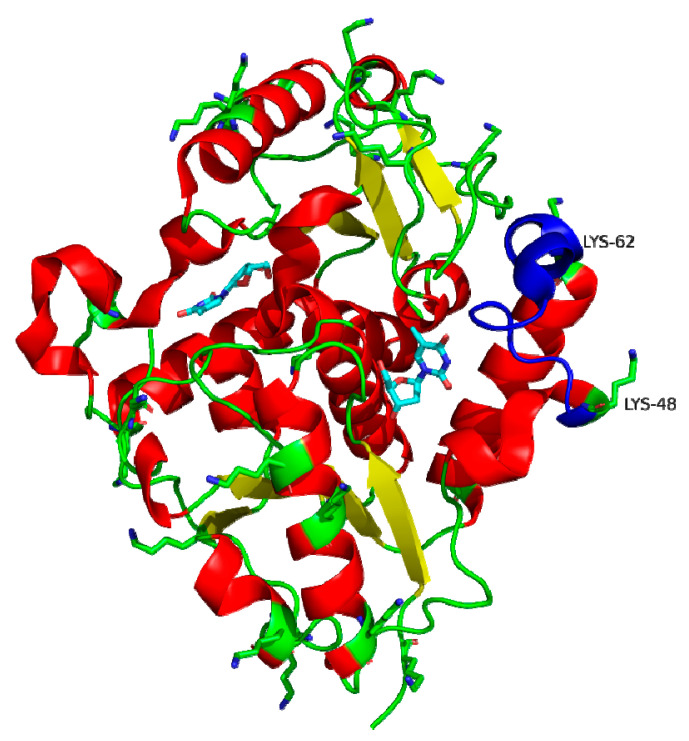
Overall representation of surface exposed Lys residues (atom colored sticks) in *Ld*NDT active site complexed with Thd (atom colored sticks). The cartoon representation is used for simplicity and ease of visualization. The catalytic loop region (amino acids 48 up to 62) is highlighted in blue. The figure has been prepared with PyMOL [34].

As previously described, *Ld*NDT is a homo-hexamer in solution, organized as a trimer of dimers [30,31]. Each dimer displays two active sites formed by different amino acids of both monomers. However, after seeing the exposed surface of lysines residues, we could observe that there are two amino acids (Lys 48 and 62) present in a catalytic loop (Figure 2). This highly mobile and flexible loop act as active site flap over, shielding it from surrounding environment [6,7], and also contains a glutamine residue (Gln46) which hydrogen bonds to N 3′and O 6′ from pyrimidine moiety.

In this context, results reported by the H++ protonation predictor program (http://biophysics.cs.vt.edu/H++, accessed on 24 March 2021) (Table S1, Supplemental Material) [48,49,50] displayed that both, Lys 48 and 62, are deprotonated at pH values required for immobilization on glyoxyl agarose (pH 10–11). As a consequence, they can be involved in the covalent linkages, reducing the mobility and flexibility of this catalytic loop and avoiding the proper binding orientation of pyrimidine ring during the catalytic process, which leads to a pronounced loss of activity.

In contrast, covalent immobilization onto SiG nanoparticles or CNBr agarose beads displayed negligible effects on enzyme activity. According to Table S1, a pKa = 7 is deduced for N-terminus, which is lined with an unipunctual immobilization through N-terminus under immobilization conditions required for immobilization in SiG nanoparticles or CNBr agarose (pH 7.0) [48,49,50,51]. Moreover, as shown in Table S1, Lys 48 and 62 residues are not deprotonated at pH 7.0, which prevents the immobilization through these surface Lys residues, which avoids the distortion of the active site architecture [6,7]. Since the N-terminus is not involved in catalysis in *Ld*NDT, and it is far from the active site, a high activity loss was not expected for the unipunctual immobilization through N-terminus (as corroborated for CNBr-*Ld*NDT, SiG-*Ld*NDT derivatives).

### 3.2. Synthesis of SiG Nanoparticles Using Several PEI Sizes 

It is known that the amount of PEI adsorbed to the surface is known as the polymer coating surface. The higher the molecular weight of the coating polymers, the larger coating nanoparticle surface is obtained and a stabilizing force of the sílica is achieved [52]. Therefore, the obtained silica nanoparticles using smaller PEI Mw could be less stabilizing than those obtained with PEI of a larger size. Based on this, PEI of three different sizes (Mw 1.200–1.300, 25.000, and 70.000) were evaluated in the synthesis of glutaraldehyde-activated biomimetic silica nanoparticles, obtaining SiG_PEI1200-1300_, SiG_PEI25000_ and SiG_PEI70000_ supports.

The immobilization of *Ld*NDT was then tested using the obtained supports and the biocatalytic capacity in cladribine biosynthesis of the derivatives SiG_PEI1200-1300_-*Ld*NDT, SiG_PEI25000_-*Ld*NDT, and SiG_PEI70000_-*Ld*NDT was compared with soluble *Ld*NDT and CNBr-*Ld*NDT (Table 1).

### 3.3. Optimization of Reaction Parameters

**Figure 3:**
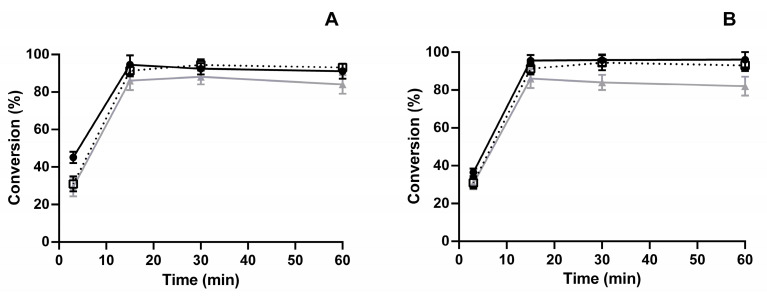
Study of several parameters using SiG_PEI25000_-*Ld*NDT derivative for cladribine biosynthesis. (**A**) Effect of pH on reaction time course, (?) 25 mM sodium acetate buffer, pH 5.0; (•) 25 mM tris-HCl buffer, pH 7.0; (▲) 25 mM tris-HCl buffer, pH 9.0. (**B**) Effect of temperature on reaction time course, (•) 30 °C, (?) 50 °C, and (▲) 60 °C.

As shown in Table 1, SiG_PEI25000_-LdNDT displayed the most interesting enzyme load/activity ratio, so we selected the derivative SiG_PEI25000_-LdNDT obtained using 230 μg of initial protein, for further experimental studies. To evaluate the effect of pH and temperature on the reaction time course, different experimental conditions were assayed (Figure 3).

According to the obtained results, neutral and low-acid pH values were shown to be the most promising experimental conditions (>90% conversion), whereas SiG_PEI25000_-*Ld*NDT displayed an optimal conversion rate in the temperature range from 30 to 50 °C. These results were similar to those previously observed for soluble protein [30].

### 3.4. SiG_PEI25000_-LdNDT Stabilization by Calcium Alginate Entrapment

Among natural matrixes, sodium alginate is considered an efficient option for enzyme immobilization because it is nontoxic, hydrophilic, biodegradable, and biocompatible [53]. It has also been demonstrated that calcium alginate entrapment significantly improves enzyme stability and allows easy recovery and reuse of the biocatalyst, favoring a subsequent bioprocess scale-up. 

With the aim of optimizing the biocatalyst for future scale-up studies, SiG_PEI 25000_-*Ld*NDT derivative was successfully entrapped in calcium alginate to obtain SiG_PEI25000_-*Ld*NDT-Alg biocatalyst [29,54,55,56]. The double stabilized biocatalyst was able to achieve 86% of cladribine conversion at 15 min, while after 30 min of reaction a conversion of 96% was achieved, equaling that achieved by its counterpart without trapping. As expected, the biosynthetic rate of the entrapped biocatalyst decreased slightly, probably due to diffusion restrictions associated with the alginate coating [54,55,56].

#### 3.4.1. Temperature and pH Stability

**Figure 4:**
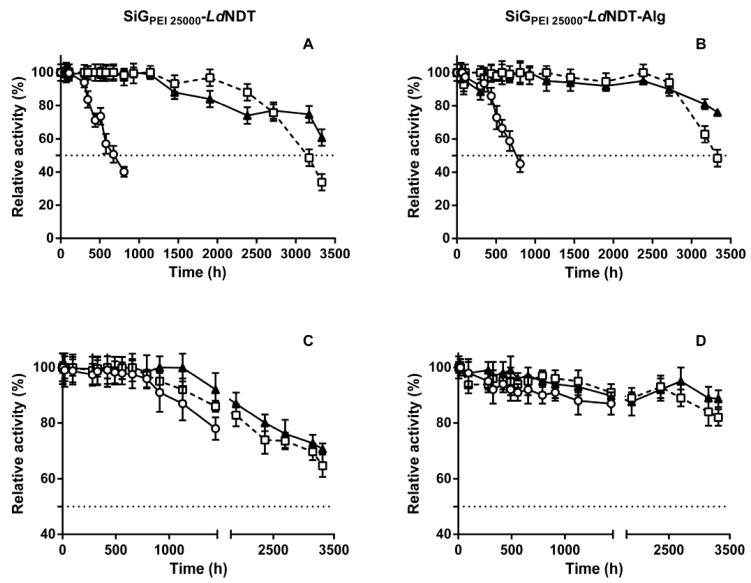
Thermal inactivation of SiG_PEI25000_-*Ld*NDT (**A**) and SiG_PEI25000_-*Ld*NDT-Alg (**B**) at different temperatures (▲) 30 °C, (□) 50 °C, and (○) 60 °C. Effect of pH on the stability of SiG_PEI25000_-*Ld*NDT (**C**) and SiG_PEI25000_-*Ld*NDT-Alg (**D**). pH 5.0 (▲), pH 7.0 (□), and pH 9.0 (○).

To determine the effect of alginate coating on biocatalysts stability, the effect of temperature and pH on the stability of SiG_PEI25000_-*Ld*NDT and SiG_PEI25000_-*Ld*NDT-Alg was assayed (Figure 4). As shown SiG_PEI 25000_-*Ld*NDT displayed excellent stability across a broad range of temperatures, exhibiting high stability at 50 °C (t_1/2_ ≈ 3100 h) and 60 °C (t_1/2_ ≈ 600 h) but also high long-term stability at 30 °C (60% retained activity) for incubation periods of more than 3300 h (Figure 4A). More interestingly, the alginate entrapped biocatalyst improved the stability of SiG_PEI 25000_-*Ld*NDT when stored at 50 °C (t_1/2_ ≈ 3300 h) and 60 °C (t_1/2_ ≈ 800 h) and enhanced the above-mentioned high long-term stability at 30 °C (≈80% retained activity during 3300 h) (Figure 4B).

In a similar way, the obtained biocatalysts, SiG_PEI25000_-*Ld*NDT and SiG_PEI25000_-*Ld*NDT-Alg, were also incubated at different pH values (pH 5.0, 7.0 and 9.0) at 50 °C (Figure 4C,D), and we could observe a similar tendency than that shown for thermal stability experiments. In this sense, both biocatalysts showed a significant improvement in stability over soluble enzymes (Figure S1), which is an operational added value. Since the primary limiting factor for industrial synthesis of purine nucleoside analogs is the poor solubility of some purine bases in aqueous solutions (e.g., adenine, hypoxanthine, or guanine) [57,58], the excellent operational stability at alkaline pHs of both derivatives qualifies them as potential biocatalysts for the industrial synthesis of purine nucleoside analogs, such as araA, araG, or ddI [5].

**Figure 5:**
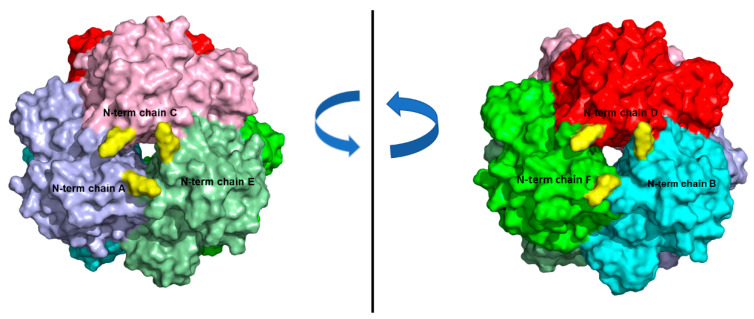
Surface representation of hexameric*Ld*NDT. The different chains (A–F) are highlighted in different colors. N-terminus residues are yellow colored.

To get a possible explanation about this phenomenon, we evaluated the disposition of N-terminus residues in both, dimeric and hexameric form, concluding that the most probably binding involves three N-terminus of different dimers located in the same axial plane (Figure 5). This three-point union provides not only higher rigidification of *Ld*NDT but also contributes to preventing subunit dissociation in the hexamer, leading to a non-expected high thermal stability. Moreover, the entrapment into calcium alginate also increased this particularly high stability, probably due to the confinement of SiG_PEI25000_-*Ld*NDT within the support, which contributes to increasingbiocatalyst stability and also protects the derivative from surrounding conditions.

#### 3.4.2. Scanning Electron Microscopy (SEM) Analysis

**Figure 6:**
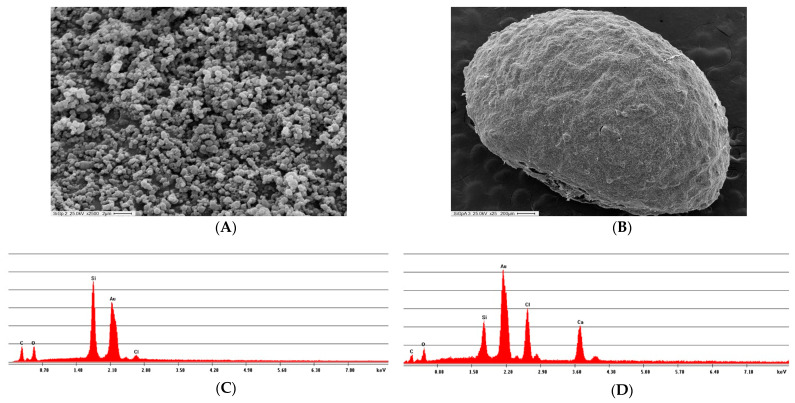
SEM and EDX analysis of SiG_PEI25000_-*Ld*NDT (**A**,**C**) and SiG_PEI25000_-*Ld*NDT-Alg (**B**,**D**) biocatalysts.

The analysis of the morphological characteristics of the SiG_PEI25000_-*Ld*NDT and SiG_PEI25000_-*Ld*NDT-Alg biocatalysts was performed by SEM (Figure 6). Typical spherical nanoparticles of biomimetic silica, in the 400–800 nm range were observed (Figure 6A). Glutaraldehyde prompts the formation of randomly agglutinated particles, but these aggregates decrease by the presence of protein, which could reduce the number of free glutaraldehyde molecules [59]. On the other hand, micrographs of SiG_PEI25000_-*Ld*NDT-Alg show spherical particles (3 mm) due to the presence of alginate (Figure 6B). Energy dispersive X-ray spectroscopy (EDX) spectra revealed the presence of calcium and chlorine on SiG_PEI25000_-*Ld*NDT-Alg, introduced by the calcium alginate matrix (Figure 6C,D).

### 3.5. Storage Stability and Reusability of SiG_PEI25000_-LdNDT and SiG_PEI25000_-LdNDT-Alg Biocatalysts

**Figure 7:**
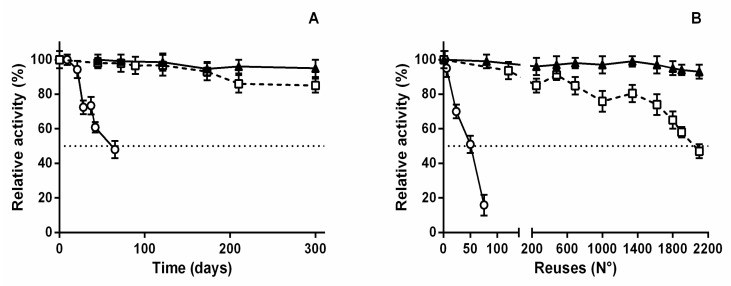
Storage stability at 4 °C (**A**) and reusability (**B**) of (▲)SiG**_PEI25000_**-*Ld*NDT-Alg, (□) SiG**_PEI25000_**-*Ld*NDT, and (○) CNBr-*Ld*NDT.

Taking into account the above mentioned results we thought SiG_PEI25000_-*Ld*NDT and SiG_PEI25000_-*Ld*NDT-Alg could be valuable biocatalysts for the synthesis of nucleoside analogs, we continued with the characterization of some other operational features, such as storage stability and reusability. Subsequently, storage stability was assayed by keeping the obtained biocatalysts at 4 °C and determining their activity at different time points (Figure 7A). Both stabilized biocatalysts maintained more than 85% of their initial activity for at least 10 months (300 days), which is far from the storage stability displayed by soluble *Ld*NDT when stored at 4 °C (85% retained activity after 30 days) [30].

Biocatalyst recycling is an essential pre-requisite for its industrial application, therefore, both biocatalysts, SiGPEI_25000_-*Ld*NDT and SiG_PEI25000_-*Ld*NDT-Alg, were employed in consecutive batch reactions (Figure 7B). Both maintained ≥50% retained activity over 1300 cycles, but at this point SiG_PEI25000_-*Ld*NDT suffered a slow but continuous decrease in retained activity. In contrast, SiG_PEI25000_-*Ld*NDT-Alg was successfully reused up to 2100 cycles with negligible loss of activity, which considerably increased the reusability of alginate-free derivative. These results are lined with those shown for thermal inactivation experiments, which displayed an excellent thermal stability, but also highlight the extremely high operational stability for both derivatives. As suggested above, the entrapment of SiG_PEI25000_-*Ld*NDT on alginate, led to a higher stabilization of the derivative but also contributed to avoiding the mechanical damage associated withmechanical stirring, which could promote the subunit dissociation and therefore a reduction in biocatalysts’ reusability.

Although a high number of NDT-mediated bioprocesses using immobilized biocatalysts have been reported during the last decade [10,11,29,32,33,38,42,43,45,46,54,55,60,61], most of them focused on cladribine synthesis [11,30,38,45,54,61], the low biocatalyst reusability under assay conditions often hinders their industrial implementation. Illustrative examples of this operational problem are cladribine synthesis using PDT from *Trypanosoma brucei* immobilized (*Tb*PDT) onto glutaraldehyde-activated microparticles (25 reuses without any activity loss, 1 mM substrates, 41% conversion) [45] or mutant *Tb*PDT_V11S_ immobilized onto Ni^2+^ chelate magnetic microparticles (10 reuses without any activity loss, 2 mM substrates, 21% conversion) [61]. This operational problem was also observed for other immobilized enzymes (e.g., cascade synthesis of cladribine using immobilized PNP and PyNP from *Geobacillus stearothermophilus*; 20 reuses, 5 mM substrates 85% conversion) [62]. Interestingly, several recent articles have addressed this issue, developing novel immobilized derivatives with high operational stability and reusability, such as *Arthrobacter oxydans cells* immobilized on alginate (20 reuses, 0.5 mM sub-strates, 85% conversion) [54] or *Thermomonospora alba* whole cells entrapped in nanostabilized hydrogels (270 reuses, 0.5 mM substrates, 89% conversion) [38]. However, these experimental results are surmounted by those shown in this work (2100 reuses, 1 mM, ≈95% conversion).

### 3.6. Bioprocess Scale-Up

Finally, as a proof of concept, the tentative scale-up of SiG_PEI25000_-*Ld*NDT-Alg mediated synthesis of cladribine was carried out using a batch system with an airlift column, increasing the biocatalyst amount and reaction medium volume by tenfold. Reaction productivity was not significantly affected in comparison with micro-scale assays, affording 90% of reaction yield at 30 min, allowing us to obtain 2.6 mg of product per hour as evaluated by HPLC. Therefore, considering its demonstrated reuse capacity, this combined biocatalyst could produce 1.8 g of cladribine, forty times more than that required per treatment cycle for an average patient (44 mg), using a dosing regimen of 0.09 mg/kg Q7D43.

Additionally, several green parameters such as E-factor, C-efficiency, and A-economy were calculated for the proposed bioprocess (Table 2)

In this way, the E-Factor value for cladribine biotransformation was <2, suggesting mass utilization efficiency and a significant decrease inwaste production. Furthermore, C-Efficiency and A-Economy are parameters used to evaluate the efficiency of synthetic reactions, showing a positive effect on atom recovery and bioprocess efficiency [64].

## 4. Conclusions

Herein we report, for the first time, the immobilization of *Ld*NDT onto different supports. Among resulting immobilized derivatives, SiG_PEI25000_-*Ld*NDT was selected as the optimal biocatalyst for further biochemical studies. With the aim to obtain a highly stable derivative, SiG_PEI25000_-*Ld*NDT was also entrapped in calcium alginate, leading to a SiG_PEI25000_-*Ld*NDT-Alg biocatalyst, which significantly enhanced the stability of soluble *Ld*NDT, but also widely outperformed the thermal stability and reusability of SiG_PEI25000_-*Ld*NDT derivative. 

Finally, the scale-up of cladribine biosynthesis by the developed biocatalyst; SiG_PEI25000_-*Ld*NDT-Alg, was achieved. Thus, an environmentally friendly process which displays an excellent stability, reusability, and high productivity for low cost cladribine biosynthesis using a novel double stabilized NDT biocatalyst, was developed. 

## Figures and Tables

**Table 1:** Covalent immobilization of *Ld*NDT onto different supports**.**

Biocatalyst	Immobilization (%)	Specific Activity (IU/mg) ^a^	Retained Activity (%)
*Ld*NDT			
230 µg ^b^	70	2.9 ± 0.1	94
SiG_PEI1200-1300_-*Ld*NDT		3.0 ± 0.1	97
15 µg ^b^	100		
95 µg ^b^	97		
230 µg ^b^	83		
470 µg ^b^	50		
SiG_PEI25000_-*Ld*NDT		3.0 ± 0.1	97
15 µg ^b^	99		
95 µg ^b^	97		
230 µg ^b^	88		
470 µg ^b^	55		
SiG_PEI70000_-*Ld*NDT		3.0 ± 0.1	97
15 µg ^b^	98		
95 µg ^b^	97		
230 µg ^b^	81		
470 µg ^b^	50		

^a^ Reaction conditions: 0.01 mg of enzyme (soluble or immobilized) were incubated at 50 °C and 200 rpm for 15 min in 1 mL 25 mM tris-HCl buffer (pH 7.0) containing 0.5 mM 2-ChlAde and 1.5 mM Thd. ^b^ Amount of added enzyme (µg). ND: non determined.

**Table 2:** Scale-up bioprocess for cladribine biosynthesis using a batch bioreactor. Environmental factors were calculated as previously reported [38,63]**.**

Specific Productivity ^a^	E-Factor	C-Efficiency	A-Economy
8.6	1.6	67	69

^a^ Cladribine (mg/h)/g catalyst.

## Data Availability

Not applicable.

## Supplementary Materials

The following are available online at https://www.mdpi.com/article/10.3390/biom11050657/s1, Figure S1: Thermal inactivation and pH effect on stability of soluble *Ld*NDT, Table S1: Computed pKa values of the surface exposed lysine residues in *Ld*NDT using the H++ server (http://biophysics.cs.vt.edu/H++, accessed on 24 March 2021).
