# Supplementary material for: Green Production of Cladribine by Using Immobilized 2′-Deoxyribosyltransferase from Lactobacillus delbrueckii Stabilized through a Double Covalent/Entrapment Technology
Source: Biomolecules. 2021 Apr 29;11(5):657. doi: 10.3390/biom11050657 (PMC8146660; doi:10.3390/biom11050657)
Supplement: Supplementary file 1 [file biomolecules-11-00657-s001.zip › Non-published material/Table S1.pdf]

Table S1. Computed pKa values of the surface exposed lysine residues in *Ld*NDT using the H++ server (http://biophysics.cs.vt.edu/H++).

N-Terminus	pKa, int*	pKa**
*Ld*NDT	7.101	6.602
**Lys (#)**	**pKa, int***	**pKa****
3	10.920	11.682
19	9.871	> 12.000
22	10.102	11.706
29	10.298	11.760
48	10.024	11.201
62	9.797	11.002
77	> 12.000	> 12.000
135	9.785	11.134
140	10.300	> 12.000
146	11.390	> 12.000

*pKa, int: computed pKa of a group assuming that there is not interaction with any other titratable group in the protein. **It corresponds to the mid-point of a titration curve (pKa ½)
