# Supplementary figures and images for: Green Production of Cladribine by Using Immobilized 2′-Deoxyribosyltransferase from Lactobacillus delbrueckii Stabilized through a Double Covalent/Entrapment Technology
Source: Biomolecules. 2021 Apr 29;11(5):657. doi: 10.3390/biom11050657 (PMC8146660; doi:10.3390/biom11050657)

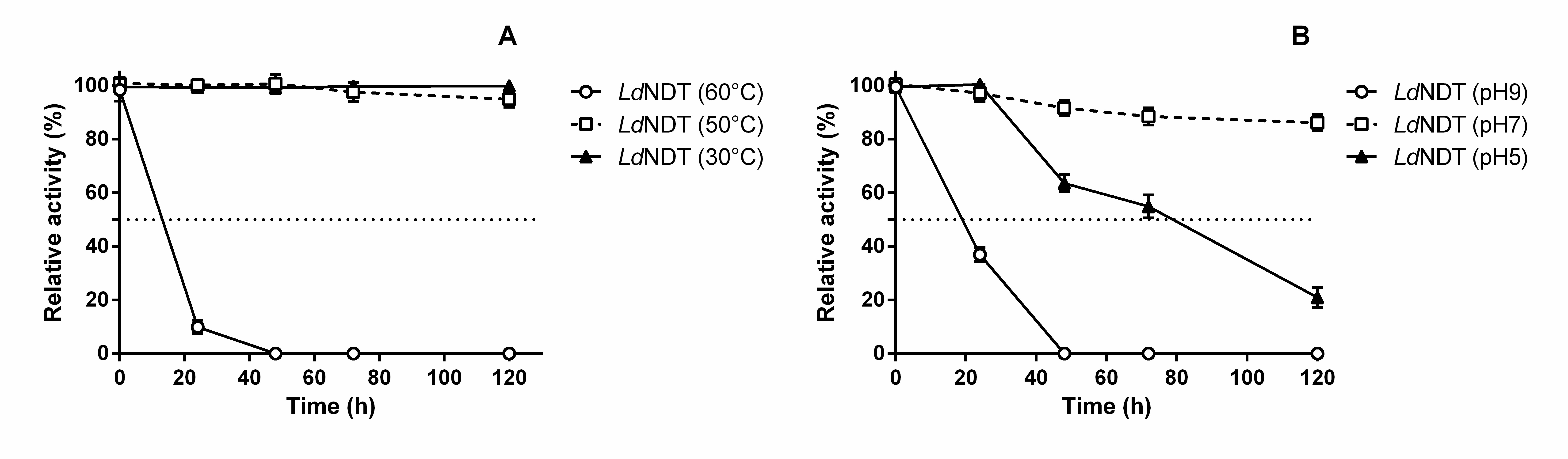

Supplement: Supplementary file 1 [file biomolecules-11-00657-s001.zip › Non-published material/Figure S1.jpg]
